# Compliance improvement in periodontal maintenance

**DOI:** 10.1590/S1678-77572010000300003

**Published:** 2010

**Authors:** Verônica Franco de CARVALHO, Osmar Shizuo OKUDA, Carlos Cheque BERNARDO, Cláudio Mendes PANNUTI, Marco Antonio Paupério GEORGETTI, Giorgio De MICHELI, Francisco Emílio PUSTIGLIONI

**Affiliations:** 1 DDS, MSc, Graduate student, Department of Stomatology, Dental School, University of São Paulo, São Paulo, SP, Brazil.; 2 DDS, MSc, PhD, Assistant Professor, Department of Stomatology, Dental School, University of São Paulo, São Paulo, SP, Brazil.; 3 DDS, MSc, PhD, Associate Professor, Dental School, University of São Paulo, São Paulo, SP, Brazil.; 4 DDS, MSc, PhD, Full Professor, Dental School, University of São Paulo, São Paulo, SP, Brazil.

**Keywords:** Periodontal diseases, Maintenance therapy, Patient compliance

## Abstract

**Objectives:**

The aim of this study was to assess the influence of efforts applied to modify the patients' behavior towards periodontal maintenance.

**Material and Methods:**

Patients were classified into three groups: Complete Compliance (participation in all visits), Irregular Compliance (irregular participation, one or more missing appointments), and Noncompliance (abandoned or never returned to the program). Complete compliers received usual procedures of the maintenance visit. The irregular compliers and non-compliers received usual procedures and strategies such as reminding next visit, informing patients on both periodontal disease and importance of maintenance, motivating the patient who showed an improvement in compliance. Thus, 137 patients were observed for 12 months.

**Results:**

The degree of compliance has increased significantly during this period (p=0.001). No association was detected between age or gender and compliance degree.

**Conclusions:**

The results have shown that the intervention applied had a favorable influence on the patients' compliance.

## INTRODUCTION

Periodontal maintenance is an integral part of periodontal therapy for patients with a history of inflammatory periodontal diseases, which starts after completion of active periodontal therapy and continues at varying intervals for the life of the dentition^1^. Inadequate control of dental biofilm may result in recolonization of the subgingival area by periodontal pathogenic microorganisms, which could compromise the results of the periodontal treatment^2,10,13,24,30^. Thus, long-term maintenance of periodontal health depends on posttreatment care. Treatment results can be maintained if etiologic factors are periodically controlled. Patients who attend regular periodontal maintenance programs have significant less attachment loss and tooth loss when compared to those who do not receive periodontal maintenance^3,4,9,11,14-16,18,21^.

The frequency of recall visits should be dictated by local, behavioral and systemic factors^1,26^. Age, smoking status, periodontal disease severity and quality of biofilm control, are factors that may increase the risk of disease recurrence^12,17,19,25,26^.

In periodontal maintenance, patients should participate actively of the treatment by both managing home biofilm control procedures and attending periodontal maintenance appointments^15,21^. However, several studies have shown that the Compliance Index (CI) for the recall visits is poor^6-8,19-23,27,29^

A previous study that evaluated patients’ adherence to the periodontal maintenance program adopted by the Postgraduate Periodontics Clinic of the Dental School of the University of São Paulo showed that only 20.2% of the patients were complete compliers, 9.0% were irregular compliers and 70.7% of the patients were non-compliers^5^. Based on these observations, some modifications were introduced in order to improve the degree of patients’ compliance. The purpose of this study was to evaluate the influence of these efforts on the improvement of patients' compliance with the periodontal maintenance program.

## MATERIALS AND METHODS

### Population

The research protocol was approved by the Research ethics Committee of the Dental School of the University of São Paulo (05/05/2003, #91/03 – 59/03).

The records of 402 out of 448 patients enrolled in the periodontal maintenance program of the Postgraduate Periodontics Clinic between March 1998 and June 2003 were reviewed. Patients who were participating in other ongoing research projects were excluded from the study (n=46).

In December 2003, 402 patients were classified in three groups, according to their compliance with the maintenance visits, before study intervention: Complete Compliance (CC) (100% compliance with the scheduled visits), Irregular Compliance (IC) (one or more missed scheduled visits), and Noncompliance (NC) (patients who abandoned the therapy or never returned to the program)^7^.

**Figure 1:**
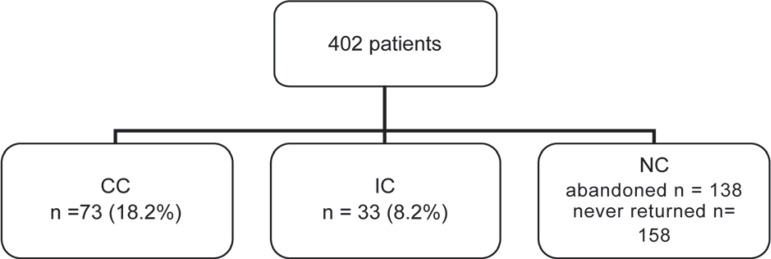
Classification of patients attending the Postgraduate Periodontics Clinic in the Complete Compliance (CC), Irregular Compliance (IC) and Non compliance (NC) groups before the beginning of the study

In the study population (n= 402), 296 (73.2%) patients were classified as NC (158 never returned to and 138 abandoned the program), 33 (8.2%) were classified as IC and 73 (18.2%) were classified as CC (Figure 1).

### Intervention

**Figure 2:**
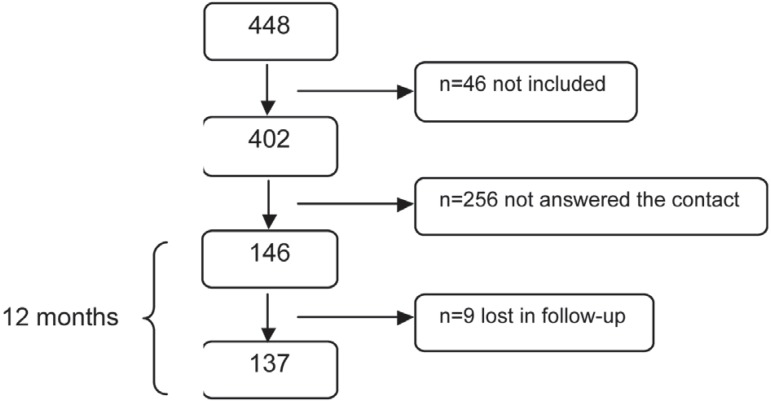
Flowchart of patient distribution

A letter containing information on periodontal disease, causes of its progression, importance of periodontal maintenance, and consequences of noncompliance was sent to the patients inviting them and stimulating their adherence to the periodontal maintenance program. From the 402 subjects, 146 answered the letters and were included in this study. All 146 participants had chronic periodontitis and were treated by postgraduate students at 3-4 months intervals of periodontal maintenance^1^. Periodontal condition is shown in Table 1. A flowchart of the patients is shown in Figure 2.

**Table 1:** Mean and standard deviation of periodontal clinical parameters: Probing Pocket Depth (PPD), Recession (R) and Clinical Attachment Level (CAL)

**Variable**	**Mean + SD**	**Min-max**
		
PPD	1.03 ± 0.76	1-9
R	1.70 ± 0.68	0-5
CAL	2.73 ± 0.63	0-10

Motivational interventions were applied^28^ during 12 months (from March 2004 to April 2005) to these 146 patients. In this period, CC subjects received the usual maintenance visit procedures, including anamnesis review, evaluation of periodontal history, radiographic examination, periodontal examination and assessment of oral hygiene status. During the maintenance visit, patients received oral hygiene instruction reinforcement, removal of supra and subgingival calculus and biofilm, crown-root polishing, and topical application of fluoride agents. At the end, another maintenance visit was scheduled or an indication for a new treatment was given, if recurrence of both clinical signs of inflammation and attachment loss were observed.

IC and NC patients were given extra motivation to increase their compliance to the treatment. A set of additional steps were used in this group, including: phone call for confirmation of the following visit, and information to the patient about periodontal disease, causes of its progression, importance of periodontal maintenance, and possible consequences of noncompliance. A single professional conducted all motivational sessions. When patients presented either better home biofilm control or regularity in their visits, a positive reinforcement was given.

## RESULTS

**Figure 3:**
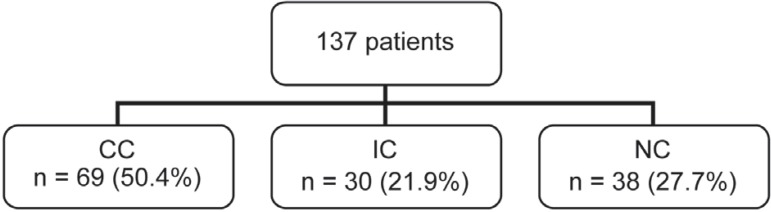
Classification of patients into the Complete Compliance (CC), Irregular Compliance (IC) and Non compliance (NC) groups before motivational intervention

Among the 146 subjects who received intervention, 9 were lost to follow-up. A hundred and thirty seven patients completed the 12-month follow-up period and were included in the statistical analysis. From these, 96 (70.1%) were women and 41 (29.9%) were men. The age of the patients ranged from 18 to 80 years (mean age of 49.8 ± 12.5 years). Before intervention, 50.4% of the patients presented CC, 21.9% presented IC, and 27.7% presented NC (Figure 3).

**Figure 4:**
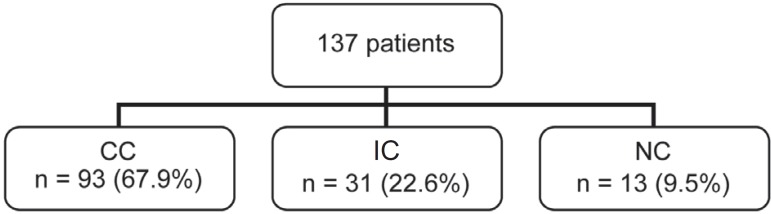
Classification of patients into the Complete Compliance (CC), Irregular Compliance (IC) and Non compliance (NC) groups after motivational intervention

After 12 months of motivational intervention, patients were reclassified into the CC, IC, NC groups, according to their responses to the intervention. Ninety-three (67.9%) patients were reclassified in the CC group, 31 (22.6%) in the IC group, and 13 (9.5%) in the NC group (Figure 4).

Differences were found among groups relative to maintenance duration before intervention (p=0.002), especially between CC and NC (p= 0.006) and between IC and NC (p=0.02) according to the test for multiple comparisons (Table 2).

**Table 2:** Distribution of patients according to their maintenance times before motivational intervention

**Groups**	**N**	Maintenance times (month) **	**P**
			
CC	69	21.3 ± 15.2	0.002 * (ANOVA)
IC	30	20.8 ± 15.1	
NC	38	10.8 ± 15.1	

* Significant difference at 5%;

** mean ± standard deviation. CC: Complete Compliance; IC: Irregular Compliance; NC: Noncompliance.

Evaluation of changes within groups in a time interval was performed with the McNemar’s test. CC and IC patients were grouped together in order to perform statistical analysis. At the beginning of the study, 99 of the 137 individuals were from either CC or IC group, and 38 were from NC group. After the motivational intervention, 124 individuals changed to either CC or IC group, and 13 changed to NC group.

Only 13 (13.1%) of the 99 subjects who were initially from CC or IC group, changed to NC. All individuals who initially belonged to NC changed to CC or IC after motivational intervention (Table 3), with significant differences (p=0.001) according to the McNemar’s test.

**Table 3:** Distribution of patients before and after motivational intervention

			**Final**	
		**CC and IC**	**NC**	**TOTAL**
				
	CC and IC	86 (86.9%)	13 (13.1%)	99 (100%)
INITIAL	NC	38 (100%)	-	38 (100%)
	TOTAL	124 (90.5%)	13 (9.5%)	137 (100%)

p=0.001 (McNemar's test; significant difference at 5%). CC: Complete Compliance; IC: Irregular Compliance; NC: Concompliance.

Association between gender, age group and cooperation degree was evaluated using chi-square test. Patients were classified in age groups according to the distribution in tertiles. There was no association between gender (0.39), age group (0.61) and final cooperation degree, that is, the cooperation degree was not shown to be higher among either men or women, or among any age cohorts (Table 4).

## DISCUSSION

In this study, a favorable modification was observed in the compliance degree of 137 patients, after 12 months of follow up. The CC group increased from 50.4% to 67.9%, IC increased from 21.9% to 22.6%, and group NC decreased from 27.7% to 9.5%. The change in the number of compliers (CC and IC) and non-compliers (NC) was statistically significant. The method used in this investigation was based on a previous study by Wilson Jr, Hale and Temple^28^ (1993), who were successful in increasing the patients’ compliance with the maintenance treatment. Those authors^28^ compared the results of two studies^28, 29^ performed with distinct populations and observed that CC increased from 16% to 32%, the number of patients with IC varied between 49% and 48%, and NC decreased from 34% to 20%. They concluded that a significant improvement occurred in CC and they were able to reduce the number of NC, with the use of motivational interventions.

The period of follow-up used herein is relatively short and thus comparable to the short duration used in other studies. More success regarding the attendance to recall appointments is reported by authors who followed their patients during a period as short as 3 years^28^. When time intervals of follow-up are longer, patients tend to show a decrease in compliance^7,23,29^. It was observed that the highest drop-out rate occurs after the first year^6,19,23,29^.

Periodontal disease has a chronic nature and its symptoms are often not sufficient to call patients’ attention. Such condition may determine that they do not consider home biofilm control and their compliance with the maintenance treatment as important^5,22^. Since a higher incidence of disregard was observed in the first years of maintenance, this period is critical for patients’ motivation^7^.

Compliance was not associated with patients’ gender in the present study, as reported by _other authors_6,8,20,23. However, an association between gender and compliance rate was shown in other studies, where women exhibited a higher compliance rate^5,8^.

All groups presented more women than men. Thus, it may be suggested that women were more interested in periodontal treatment. According to Demirel and efeodlu^8^ (1995), the fact that most women in Istanbul do not have a formal occupational labor and thus have more free time to take care of their health could have accounted for the obtained results. Demetriou, Tsami-Pandi and Parashis^7^ (1995) stated that Greek women showed a higher compliance with the treatment because they are more concerned about their appearance and afraid of losing their teeth. Furthermore, they either do not have a formal occupational labor or have a part-time job, which means, according to them, more free time and less stress. Offering the patients dental appointments that do not coincide with their working hours could be an efficient strategy to meet the needs of people who cannot be absent from their work^8,22^. Up to now, this alternative is not available for our patients.

Regarding patients’ age, no significant differences were observed among CC, IC, and NC groups. Most studies show that elderly patients are the best compliers^20,23^. Since younger patients have more financial difficulties and are usually under more pressure in their jobs, dental preservation is not ranked in their priority list^20^. Interestingly, the older patients, the higher the compliance to the periodontal maintenance programs.

## CONCLUSIONS

It may be concluded that the efforts applied in this study had a significant favorable influence on the patients’ behavior regarding their compliance with the periodontal maintenance treatment. Although favorable results could be achieved in the present study, the conclusions derived from them are limited to a short-term follow-up of patients and should not be extended to a long-term period. It is believed that long-term studies are needed to allow both a better understanding of patients’ behavior undergoing maintenance treatment and the elaboration of procedures with higher efficacy and motivation.
